# Revisiting the Mucosa of the Gastric Cardia: A Scope to Modify an Upper Gastrointestinal Endoscopy

**DOI:** 10.7759/cureus.42443

**Published:** 2023-07-25

**Authors:** Sudipta D Baruah, Bishwajeet Saikia, Raktim P Tamuli, Bipul K Das

**Affiliations:** 1 Anatomy, North Eastern Indira Gandhi Regional Institute of Health and Medical Sciences, Shillong, IND; 2 Forensic Medicine, Gauhati Medical College and Hospital, Guwahati, IND; 3 Pediatrics, Tezpur Medical College and Hospital, Tezpur, IND

**Keywords:** carcinoma esophagus, adenocarcinoma, endoscopy, barrett’s esophagus, gerd, cardiac mucosa, gastroesophageal junction, gastric cardia

## Abstract

Introduction

The mucosa in the cardiac region of the stomach has been less understood. Cardiac mucosa (CM) with less parietal and oxyntic cells has been defined as a normal mucosa. Studies have shown that CM can be the result of occult reflux. Oxyntic mucosa (OM) is normal, and it changes to CM with age. In advancing age, it is more common to find CM instead of OM and oxyntocardiac mucosa (OCM). This study is an attempt to examine the distribution of the three different types of mucosa in various age groups.

Materials and methods

The study was conducted in the Department of Anatomy and Department of Forensic Medicine and Toxicology of Gauhati Medical College, Guwahati, Assam, India, from 2017 to 2019. Once the stomach was opened, histological specimens were prepared, and the type of mucosa was observed and recorded. Then, the distribution of the types of mucosa in various age groups was analyzed.

Results

The distribution of mucosa varies significantly across different age groups, and CM increases with age.

Conclusion

Our present study suggests that CM frequency increases with age. This is in accordance with studies that suggest that CM is a result of occult reflux with age. This observation creates a scope to revise the approaches for upper gastrointestinal (GI) endoscopy.

## Introduction

The gastric cardia, a term that has been commonly used in the scientific and academic literature, has never been clearly defined [1]. Ellis et al. referred cardia as the extreme lower end of the esophagus [2]. Endoscopically, the most proximal part of the stomach before the appearance of the gastric rugal folds is considered the gastric cardia [3]. Apart from the several confusions in gross anatomy, there are differences in opinions regarding the nature of mucosa present in this region [4]. It has been suggested that the beginning of the typical oxyntic mucosa (OM) of the stomach should be taken as the true gastroesophageal junction (GEJ) for histopathological purposes and not the squamocolumnar junction (SCJ) marked by the Z line [5]. Studies have stated that normally the cardiac region should have only two types of mucosa, namely, the stratified squamous epithelium (SSE) of the esophagus above the Z line and OM below the Z line. With age, the OM just below the Z line is replaced by a variable amount of mucous-secreting pure cardiac mucosa (CM) or oxyntocardiac mucosa (OCM) with or without intestinal metaplasia (IM) [6]. The change of OM to OCM or CM happens due to repeated reflux of the acid content of the stomach, which some authors consider to be a pathological change [7,8]. Furthermore, it has been well established that gastroesophageal reflux (GER) increases with increasing age [9]. In light of the above viewpoint, it has been suggested that there would be an increased distribution of OCM and CM among the population with increasing age [7]. The view described above is very different from the widely accepted classical view of the cardiac region suggested by Hayward [1]. Hayward stated that CM is a normal histological feature of the gastric cardia for an area about 1-2 cm distal to the Z line where OM begins [1,6].

The association between GER and adenocarcinoma is well established [10]. OM is comparatively more resistant to the effect of gastric juices than CM and OCM [7]. When the more resistant OM is replaced by the less resistant CM or OCM, this area becomes more prone to the detrimental effects of GER [6]. In this study, we attempted to examine the types of mucosa in the gastric cardia in 50 specimens and their distribution across various age groups.

## Materials and methods

Our study was conducted in a tertiary care institution in Guwahati, Assam, India, from 2017 to 2019. Due clearance for the study was obtained from the Institutional Ethics Committee of Gauhati Medical College and Hospital, with approval number MC/190/2007/Pt-1/EC/134. Cadaveric dissection was performed at the Department of Anatomy, and autopsy specimens were collected from the Department of Forensic Medicine and Toxicology. A total of 50 specimens were subsequently obtained, 32 male and 18 female.

Inclusion criteria

Individuals with deaths due to road traffic accidents or cardiac failure were included in this study.

Exclusion criteria

Cadavers with significant pathology (clinical history/gross examination) involving the stomach and esophagus (upper gastrointestinal (GI) surgery) were excluded.

The age and sex of the deceased were noted. The relevant cause of death and any significant clinical history were ascertained from death certificates and confirmed from the nearest relatives. Informed consent for the study was also obtained from the nearest relatives.

Dissection and tissue fixation

**Figure 1:**
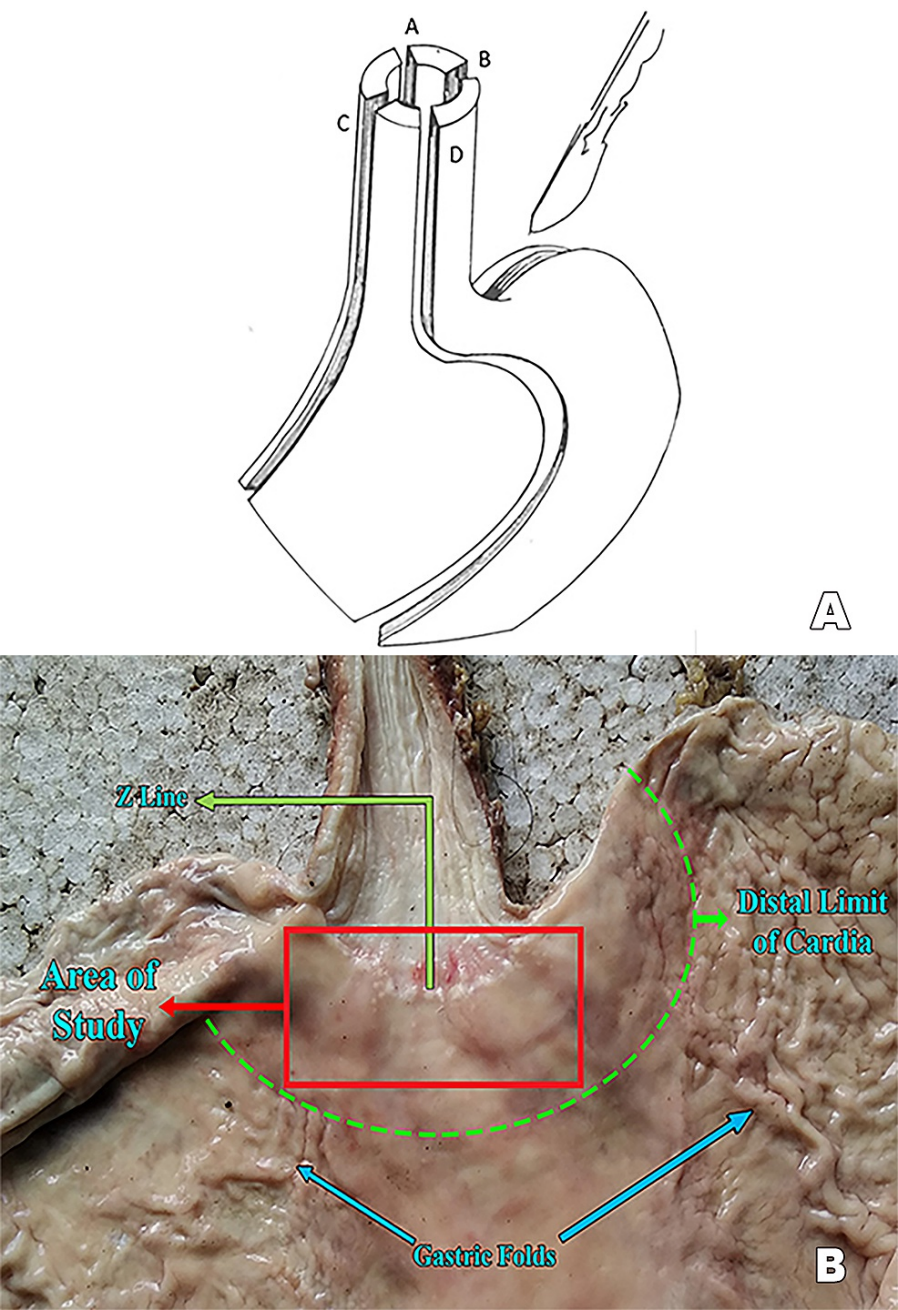
A: Illustration of incisions to open the stomach. In this study, we used incision B. B: Anatomy of the area of study.

The stomach was cut open from the pylorus along the greater curvature (Figure 1a). The incision was extended to the esophagus. Once opened, the specimen was laid flat on a flat board. The gastric cardia was the area chosen for this study (Figure 1b).

The distal demarcation of the gastric cardia was taken as the beginning of the gastric folds. The specimen was cut into thin pieces of approximately 5 to 8 mm in thickness. The tissue was fixed in 10% formal saline (10% formal saline = 100 mL formalin + 900 mL tap water + 8.5 g sodium chloride) for a period of 24 to 48 hours.

The tissue was then subjected to dehydration by immersing them in increasing strengths of alcohol (50%, 70%, and 90%) and finally in absolute alcohol for specified times. The tissue was then placed in xylene for a period of 30 minutes.

Wax impregnation of the tissue was done by using a liquid paraffin wax bath. The tissue was passed through the liquid paraffin maintained at a temperature of about 60ºC. Paraffin blocks were prepared using Leuckhart’s L-Blocks. The blocks were cleaned with glycerine. Molten paraffin was poured into squares of appropriate sixes, and hot-tipped forceps were used to dip the tissues from the wax impregnation bath. Small strips of paper were used to label the wax blocks, which were dipped into the blocks before they solidified. The wax blocks were solidified by submerging them in a cold water bath.

The paraffin blocks were then cut at 5 µm thickness using a rotary microtome. The sections were floated on lukewarm water in a floatation bath at 37ºC. This stretches the wax strips and removes the folds. The strips were cut into pieces of desired sizes using a needle dipped in xylene. The sections were attached to cleaned glass slides using egg albumin and dried on a hot plate of slide warmer boxes. The sections were stained by routine hematoxylin and eosin staining. At the end of the staining process, the sections were dehydrated in ascending strengths of alcohol, cleared with xylene, and mounted with dibutylphthalate polystyrene xylene (DPX).

**Figure 2:**
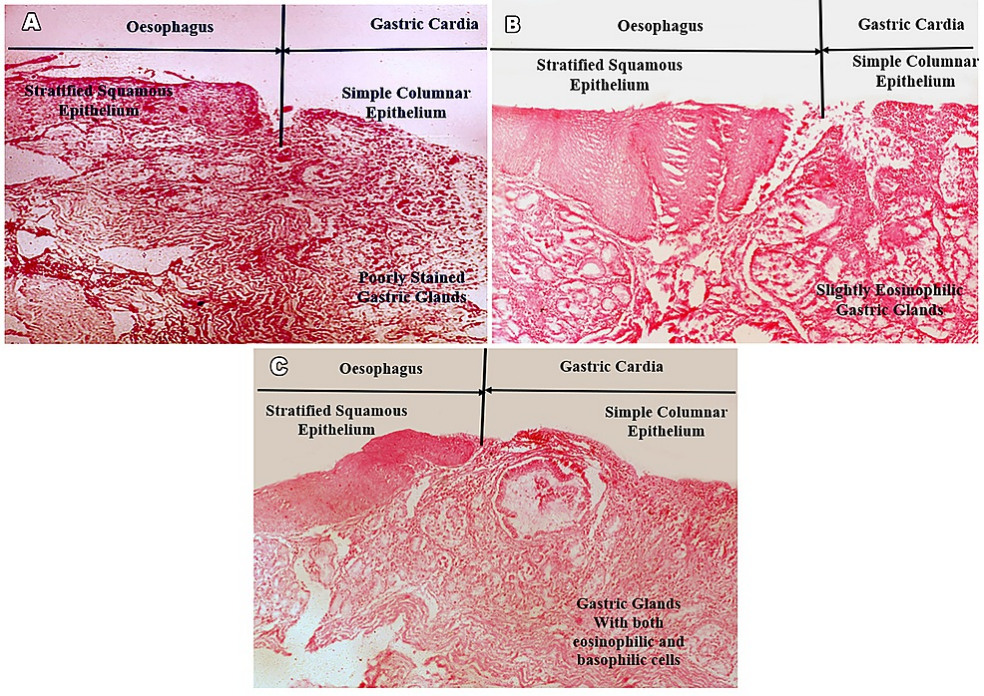
A: Cardiac mucosa (CM) with characteristic poorly staining gastric glands; stain: hematoxylin and eosin; Magnification: 10X. B: Oxyntocardiac mucosa (OCM) with slightly eosinophilic gastric glands; stain: hematoxylin and eosin; magnification: 10X. C: Oxyntic mucosa (OM) with characteristic mixed staining gastric glands; stain: hematoxylin and eosin; magnification: 10X.

The stained sections were observed under a light microscope under both low-power (10X) and high-power (40X) magnifications. The characteristics of the glands were noted on the columnar CM. The types of mucosa of the gastric cardia were classified under three groups based on the type of cells in the epithelium: 1) CM: predominantly mucous-secreting cells; mucosa poorly stained in hematoxylin and eosin stain (Figure 2a). 2) OCM: mucous cells and parietal cells in equitable distribution; mucosa slightly eosinophilic in hematoxylin and eosin stain owing to scattered parietal cells (Figure 2b). 3) OM: predominantly oxyntic cells with scattered parietal cells with scanty mucous cells; mucosa shows equitable staining of both acidic and basic dye in hematoxylin and eosin stain (Figure 2c).

## Results

**Figure 3:**
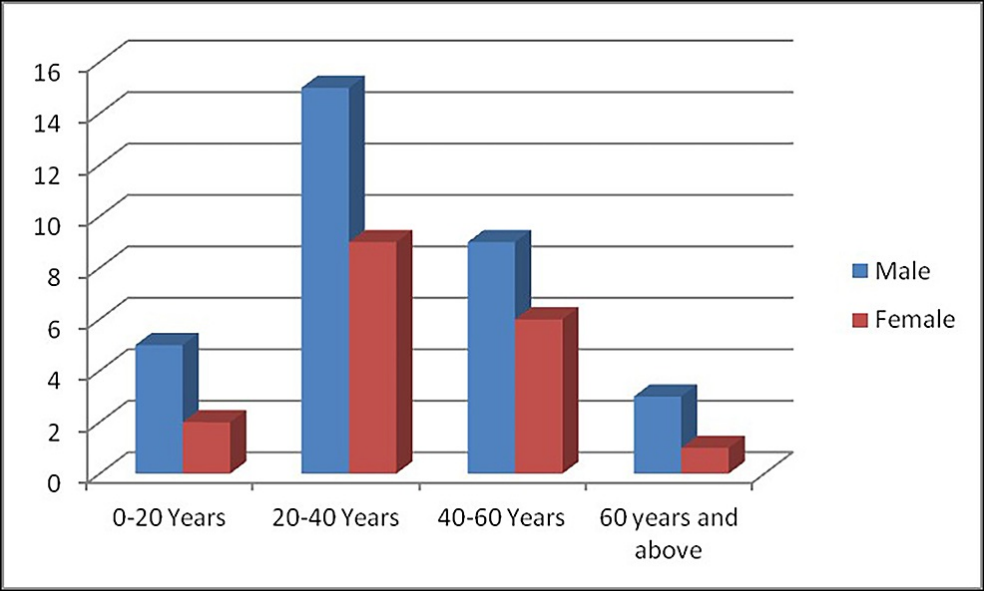
Distribution of specimens as per age and gender.

The data were collected, observed, and statistically evaluated in age intervals of 20 years for both male and female (Table 1 and Figure 3).

**Table 1:** Distribution of specimens as per age and gender.

Groups	Male	Female
0-20 years old	5	2
20-40 years old	15	9
40-60 years old	9	6
60 years old and above	3	1
Total	32	18
Grand total	50	

**Figure 4:**
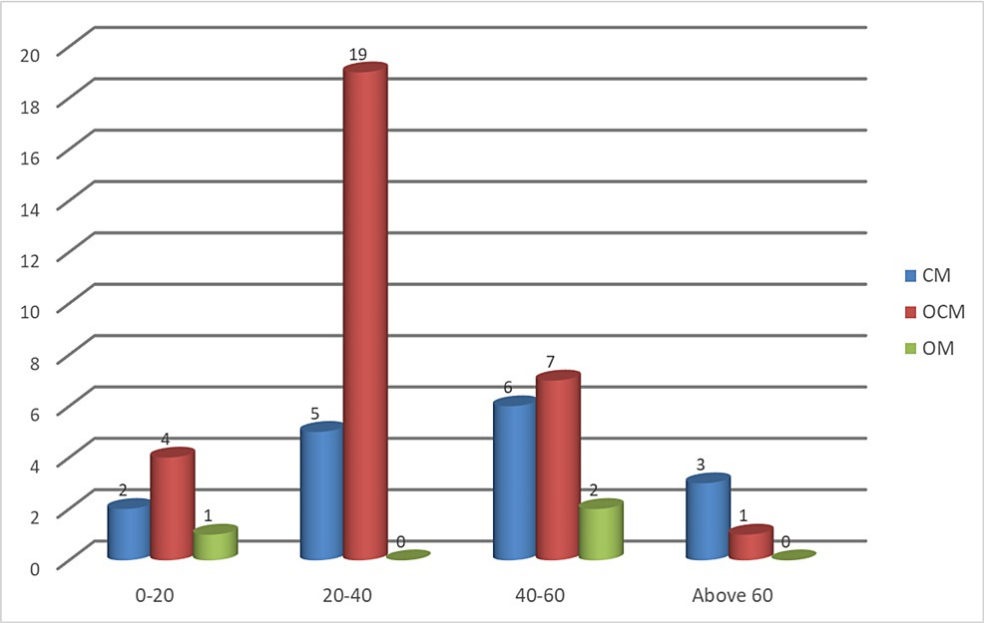
Distribution of different types of mucosa of the gastric cardia in various age groups. CM: cardiac mucosa; OCM: oxyntocardiac mucosa; OM: oxyntic mucosa

The mucosa of the gastric cardia was observed, classified, and recorded based on the type of glands (Table 2 and Figure 4).

**Table 2:** Distribution of different types of mucosa of the gastric cardia in various age groups. CM: cardiac mucosa; OCM: oxyntocardiac mucosa; OM: oxyntic mucosa

Mucosa of the gastric cardia			
Age group	CM	OCM	OM
0-20 years old	2	4	1
20-40 years old	5	19	0
40-60 years old	6	7	2
Above 60 years old	3	1	0
Total	16	31	3

For testing the presence of any dependence between distributions of mucosa across the different age groups, the Fisher’s exact test was used.

The null hypothesis is H_0_: The distribution of mucosa is independent of the different age groups.

The p-value of the test is 0.0269 < 0.05, which is significant at 5% level of significance. Thus, we reject H_0_ at 5% level of significance and concluded that the distribution of different types of mucosa of the gastric cardia varies significantly across the different age groups.

## Discussion

The study findings of different authors for the histology of the gastric cardia are tabulated with the findings of our study (Table 3).

**Table 3:** Comparison of the findings of previous authors with the current study for the mucosa at the gastric cardia

Sl No	Author	Type of mucosa		
		Cardiac mucosa	Oxyntocardiac mucosa	Oxyntic mucosa
1	Chandrasoma et al. [7]	44%	56%	-
2	Shi et al. [11]	20.12%	36.05%	36.56%
3	Kim et al. [12]	66.7%	33.3%	-
4	Present study	32%	62%	6%

Chandrasoma et al. found that the gastric cardia contains OM in most specimens (56%) [7]. In the remaining 44%, pure CM was found. Shi et al. found pure CM in 20.12% of specimens and OCM in 36.05% of specimens [11]. In the remaining 36.56%, they found OM. Kim et al. found CM in 66.7% of specimens and OCM in 33.3% of specimens [12].

In the present study, 62% of the specimens were found to have OCM. CM was found in 32% of the specimens. In the remaining 6%, OM was found. Our findings correlated with those of Chandrasoma et al. and Shi et al. [7,11].

A significant variation was found in the Fisher’s exact test done for the distribution of the different types of mucosa across various age groups (p-value < 0.05). In all the age groups, most of the specimens showed OCM. However, CM was found in all of the specimens collected from the cadavers above 60 years of age.

A recent view suggest that the presence of CM in the gastric cardia should not be considered as a normal feature. This view relates to the development, as embryologically there is a stratified squamous epithelium in the esophagus up to the Z line and then the typical gastric mucosa, OM, begins at the Z line. With age, due to repeated exposure of the gastric acid, the mucosa in the cardiac region gradually changes to the predominantly mucous-secreting CM. As a result, the most proximal appearance of a typical gastric mucosa, OM, shifts further away from the Z line, creating an intermediate zone. This resultant intermediate zone gradually transforms into OCM and then to CM due to the continued exposure to gastric juices. Endoscopy-based studies have also found that CM is not a usual finding in the cardiac region, even adjacent to the Z line [5,8,13,14].

In our study, OM and OCM were found more in the gastric cardia of young adults. Only 20% of mucosa was found to be CM in the 20-40-year-old age group. With increasing age, the distribution of CM also increases. In the 40-60-year-old age group, about 40% of the mucosa was CM. In the age group of 60 years old and above, all the specimens that were observed were found to contain CM. This correlates to the findings of Jain et al. and Oberg et al., as discussed above [13,14].

The accepted criterion for the diagnosis of Barrett’s esophagus (BE) is the presence of IM in the lower end of the esophagus. This is further divided into short-segment and long-segment BE. Anatomically, the area assessed for this is between the most proximal gastric rugal folds and the Z line [15,16]. Currently, patients who have OCM, CM, or OM in this region are termed as normal. However, there is increasing evidence that CM and OCM in this region are results of repeated reflux esophagitis with increasing age and therefore cannot be considered as normal [8]. There is also added evidence that the presence of CM is very frequently associated with chronic inflammation of this region, which is considered to be a result of occult reflux [17]. All of these observations further suggest that the existing criteria for gastroesophageal reflux disease (GERD) and BE may be revisited and revised. In fact, some authorities have suggested that the inflammation of the gastric cardiac region and presence of CM in this region should be considered as reflux esophagitis and a result of metaplasia of the esophagus. It was also proposed that everything proximal to the gastric rugal folds is esophageal and thus any lesion or tumor of this area should be regarded as pathologies of the esophagus [4].

Limitations of our study

A cadaveric study like ours might not be representing the gastric cardia in live subjects. Correlating the study with endoscopic studies of the gastric cardia can establish further evidence. With a bigger sample size, the study can have a better impact.

## Conclusions

The acknowledgement of CM as an abnormal structure resulting from GER with age possibly calls for a re-evaluation of our entire outlook toward GERD, BE, and their associated risk toward the development of adenocarcinoma at the lower end of the esophagus. Furthermore, screening GERD patients for “OM to CM transformation” and categorizing them as high risk for intestinal metaplasia, BE, and adenocarcinoma may be helpful in decreasing the global morbidity and mortality caused by adenocarcinoma. The present approach considers the patients too normal if there are no apparent sign of reflux in endoscopy regardless of whether the type of mucosa is CM, OCM, or OM. Therefore, we suggest that the endoscopic approach can be modified to take a routine biopsy of the mucosa near the Z line in these patients to check for CM.
